# Drug Repurposing for Glioblastoma and Current Advances in Drug Delivery—A Comprehensive Review of the Literature

**DOI:** 10.3390/biom11121870

**Published:** 2021-12-13

**Authors:** Safwan Alomari, Irma Zhang, Adrian Hernandez, Caitlin Y. Kraft, Divyaansh Raj, Jayanidhi Kedda, Betty Tyler

**Affiliations:** Hunterian Neurosurgical Research Laboratory, Department of Neurosurgery, Johns Hopkins School of Medicine, Johns Hopkins University, Baltimore, MD 21231, USA; salomar1@jhmi.edu (S.A.); irma.zhang@gmail.com (I.Z.); aherna47@jhu.edu (A.H.); caitlin.y.kraft@gmail.com (C.Y.K.); draj3@jhmi.edu (D.R.); jkedda@jhmi.edu (J.K.)

**Keywords:** brain tumor, drug delivery, glioma, repurposed drugs

## Abstract

Glioblastoma (GBM) is the most common primary malignant brain tumor in adults with an extremely poor prognosis. There is a dire need to develop effective therapeutics to overcome the intrinsic and acquired resistance of GBM to current therapies. The process of developing novel anti-neoplastic drugs from bench to bedside can incur significant time and cost implications. Drug repurposing may help overcome that obstacle. A wide range of drugs that are already approved for clinical use for the treatment of other diseases have been found to target GBM-associated signaling pathways and are being repurposed for the treatment of GBM. While many of these drugs are undergoing pre-clinical testing, others are in the clinical trial phase. Since GBM stem cells (GSCs) have been found to be a main source of tumor recurrence after surgery, recent studies have also investigated whether repurposed drugs that target these pathways can be used to counteract tumor recurrence. While several repurposed drugs have shown significant efficacy against GBM cell lines, the blood–brain barrier (BBB) can limit the ability of many of these drugs to reach intratumoral therapeutic concentrations. Localized intracranial delivery may help to achieve therapeutic drug concentration at the site of tumor resection while simultaneously minimizing toxicity and side effects. These strategies can be considered while repurposing drugs for GBM.

## 1. Introduction

Glioblastoma (GBM) is the most common malignant primary brain tumor in adults. The current standard of care is surgical resection, radiotherapy and chemotherapy. However, tumor recurrence is nearly universal. Median overall survival remains between 12 and 15 months, with a 5-year survival rate of less than 5% [1,2]. Currently, temozolomide (TMZ) is the mainstay for chemotherapy treatment. However, since FDA approval of TMZ in 2005, half of the patients with GBM have been nonresponsive to TMZ-treatment due to high expression of O6-methylguanine methyltransferase (MGMT) [3]. Moreover, in vitro studies have demonstrated a corresponding significant increase of GBM stem cells (GSCs) within the total GBM cell population after treatment with TMZ [4]. This resistance has generated a compelling need for innovative and effective therapeutic strategies for patients with GBM.

Drug repurposing is a concept in which previously approved drugs are used for new indications other than their traditional use. There has been growing interest in drug repurposing in many fields of medicine [5,6]. Repurposing drugs has several advantages over novel drug discovery. Since these drugs have already undergone the rigorous process of investigation of safety profiles and pharmacokinetic properties [7], drug repurposing can be significantly less costly and less time intensive than novel drug discovery [8]. Here we review different aspects of drug repurposing for GBM therapeutics, including limitations and recent advances to overcome these limitations. Since many of these drugs are at various stages of investigation, we classified them into two groups: drugs in the clinical trial phase (Table 1) and drugs undergoing pre-clinical testing (Table 2).

## 2. Drugs under Clinical Investigation

### 2.1. Drugs Used for Other Central Nervous System Disorders

Drugs of the nervous system are prescribed to treat a wide range of disorders including neurodegenerative diseases, psychiatric illnesses, headaches, epilepsy, and autoimmune diseases among other pathologies [44]. Despite the wide range of mechanisms of their action, as well as possible side effects with extended use, there are key qualities that make these drugs appealing candidates for GBM therapy. The blood–brain barrier (BBB) is one of the significant obstacles that must be overcome when considering drugs for GBM treatment. Because drugs used in nervous system disorders have already been shown to cross the BBB in therapeutic concentrations, this challenge has already been addressed [45,46]. Incidentally, there have been reports of lower GBM incidence among patients taking depression and schizophrenia drugs, which has suggested potential antineoplastic properties of these drugs [45,47,48,49]. In addition, these drugs are widely available and can serve a dual purpose for GBM patients. Reports have estimated that around 90% of GBM patients suffer from comorbid psychiatric disorders and are already treated with one or more drugs within this class [50,51]. Therefore, it is not surprising that various drugs in this class are currently undergoing clinical trial investigation for GBM treatment. In this section, we provide a brief review of literature on these drugs, their antineoplastic mechanisms of action, and results of published studies.

### 2.2. Memantine

Memantine is an N-methyl-D-aspartic acid (NMDA) antagonist that is used to treat a number of neurological conditions, most notably mild to moderate Alzheimer’s disease [52]. Through activation of the NMDA receptor, the neurotransmitter glutamate not only has trophic functions within the mammalian central nervous system (CNS) but is also suggested to have similar proliferative effects in cancer cells [53]. Additionally, glutamate is known to accumulate in the fluid surrounding tumor cells and triggers glutamate receptor activation. This can lead to excitotoxicity and neuronal cell death, which may provide a favorable environment for GBM tumor cells to invade into its surroundings [54]. Anti-NMDA drugs such as memantine may reduce glutamate action and protect the neurons surrounding tumor cells from inflammation and excitotoxicity [54]. In fact, in vitro studies demonstrate that glutamate antagonists enhance cell death, alter morphological features of tumor cells, and inhibit migration and division of tumor cells [55].

In a Phase I clinical trial conducted by Maraka et al., 85 patients diagnosed with GBM were randomized into several treatment arms, including combination therapy of TMZ, memantine, mefloquine, and metformin. Combination treatments were administered as post-radiation adjuvant therapy [9]. The primary endpoint of the study was the occurrence of dose-limiting toxicity. The study successfully demonstrated the feasibility in combining TMZ with mefloquine, metformin, and memantine. These drug combinations were shown to be generally well tolerated. The study also established the doses of each drug that could be safely used in combination with TMZ. The dose-limiting toxicities included dizziness (memantine) and gastrointestinal effects (metformin). The progression-free survival (PFS) rate at 6 months was 50% (95% CI, 40–63%), the median overall survival (OS) was 21 months (95% CI, 16.2–29.7 months), and the 2-year survival rate was 43% (95% CI, 34–56%) [9]. However, Maraka et al. noted significant limitations of this study. Since it was a single-institution Phase I trial, the study was not designed to assess efficacy of the drug [9]. Therefore, future Phase II trials with larger patient cohorts are encouraged to evaluate the clinical benefit and efficacy of memantine.

### 2.3. Levetiracetam

Levetiracetam is an anticonvulsant commonly prescribed to brain cancer patients to prevent or treat focal seizures [8]. It enhances gamma-aminobutyric acid (GABA) release and has a relatively high therapeutic index in the CNS when compared to other anti-epileptic drugs [56]. Levetiracetam may have potential anti-GBM properties as well. In 2016, Peddi et al. reported a case of GBM regression after a patient received seizure prophylaxis treatment which contained dexamethasone and levetiracetam [57]. Reports have theorized that levetiracetam can enhance expression of p53, increase binding of histone deacetylase-1 (HDAC1) complex to the MGMT promoter complex, and sensitize GBM cells to TMZ [58].

To investigate the efficacy of levetiracetam as a chemosensitizer to TMZ, Kim et al. retrospectively reviewed 103 consecutive patients with primary GBM who underwent surgery and postoperatively received concomitant chemoradiotherapy (CCRT) and adjuvant chemotherapy with TMZ. Fifty-nine patients (56%) of this cohort received levetiracetam while receiving TMZ for at least 3 months [10]. Cox-regression survival analysis revealed that the median PFS and OS for patients who received levetiracetam in combination with TMZ (median PFS: 9.4 months; median OS: 25.7 months) were significantly longer than those for patients who did not receive levetiracetam (median PFS: 6.7 months; median OS: 16.7 months; *p*  =  0.010 and *p*  =  0.027, respectively). This suggested that the levetiracetam served as a sensitizer for TMZ during the adjuvant therapy period [10].

The authors of this study noted several limitations. Due to the retrospective nature of the study, selection bias may have influenced the results [10]. Confounding factors such as age, seizure incidence, and the impact of surgical resection on seizure incidence may have influenced the survival outcomes as well [10]. Finally, long-term follow-up was not performed for the group of patients receiving levetiracetam. Prospective randomized clinical trials are needed to further assess the survival benefit of levetiracetam as a potential chemosensitizer in patients with GBM [10].

### 2.4. Valproic Acid

Similar to levetiracetam, valproic acid is an antiepileptic drug commonly used in patients with brain tumors [10]. There have been incidental reports that patients with GBM taking valproic acid had better survival outcomes compared to patients who either did not take valproic acid or took other antiepileptic drugs [59,60,61]. Valproic acid demonstrates a potential antineoplastic effect through its ability to inhibit histone deacetylases (HDAC) [8]. HDACs promote neoplasia by altering transcription of tumor suppressor genes and are often overexpressed in GBM cells [8,62]. Through HDAC inhibition, valproic acid may also increase tumor cell sensitivity to ionizing radiation while sparing healthy cells from radiation damage [63]. Of note, valproic acid has been used in combination with other drugs such as TMZ, topoisomerase, and carboplatin for studies investigating treatment efficacy against GBM and medulloblastoma [64]. More recently, numerous clinical trials are currently investigating its efficacy against GBM.

An open-label Phase II clinical trial conducted by Krauze et al. evaluated the safety, tolerability, and efficacy of concomitant RT/TMZ therapy with high-dose valproic acid, followed by adjuvant TMZ for patients diagnosed with GBM [11]. The primary objectives of the study were 6-month PFS and OS. Thirty-seven patients were treated with concomitant valproic acid with RT and TMZ. OS was 29.6 months (range: 21–63.8 months), and median PFS was 10.5 months (range: 6.8–51.2 months). OS at 6, 12, and 24 months was 97%, 86%, and 56%, respectively. PFS at 6, 12, and 24 months was 70%, 43%, and 38% respectively. Of note, while 6-month PFS was also similar to other clinical trials investigating aforementioned drug combinations, the median OS of this trial was 29.6 months, which was markedly prolonged compared to the median survival of previous studies which ranged between 8.6 and 19.3 months. The most common toxicities noted with the combination therapy were blood and bone marrow toxicity (32%), neurological toxicity (11%) and metabolic and laboratory toxicity (8%) [11].

Another Phase II study by Su et al. investigated the efficacy of concomitant valproic acid and RT followed by valproic acid and bevacizumab therapy in children diagnosed with high grade gliomas (including GBM, anaplastic astrocytoma, or gliosarcoma) and diffuse intrinsic pontine glioma (DIPG) [12]. The primary endpoint of the study was one-year event-free survival. Twelve of the thirty-eight children recruited for this study were diagnosed with GBM [12]. Median event-free survival (EFS) and OS for DIPG were 7.8 (95% CI 5.6–8.2) and 10.3 (7.4–13.4) months, and estimated one-year EFS was 12% (2–31%). Median EFS and OS for high grade glioma were 9.1 (6.4–11) and 12.1 (10–22.1) months and estimated one-year EFS was 24% (7–45%). Although event-free survival and overall survival for both DIPG and GBM cohorts were not improved compared to previous studies, partial responses and a high rate of pseudo progression were noted in both the DIPG and high grade glioma group [12]. Similarly, as noted above, when valproic acid was combined with RT, high toxicities required discontinuation and modification of the dosing of the valproic acid due to thrombocytopenia, fatigue, and hypertension. Su et al. proposed that the small sample size and the heterogeneity of the high-grade glioma group likely prevented the detection of significant improvement in survival curves and the authors suggested that there is potential use for valproic acid as an enhancer for radiation.

### 2.5. Disulfiram

Disulfiram is used to treat alcohol abuse by inhibiting liver acetaldehyde dehydrogenase (ALDH) and preventing the enzyme from catalyzing the oxidation of acetaldehyde to acetate. If a patient taking disulfiram imbibes alcohol, acetaldehyde accumulates in the body and causes aversion symptoms reminiscent of a hangover [8]. Various in vitro and in vivo studies have demonstrated efficacy against GBM cells, and several mechanisms have been proposed. Studies found that ALDH is upregulated in tumor cells that exhibit growth enhancement and resistance to chemotherapy treatment [65]. Thus, inhibition of ALDH may potentially inhibit GBM tumor growth. Additionally, diethyldithiocarbamate, a metabolite of disulfiram, chelates with copper and zinc ions to form complexes that inhibit proteasomes and increase cytotoxicity through accumulation of oxygen free radicals [66,67]. These properties make disulfiram a promising drug to be repurposed for GBM treatment.

Huang et al. conducted a Phase I open-label, single-arm, dose-escalation and dose-expansion study for disulfiram in combination with adjuvant TMZ for patients with newly diagnosed GBM [13]. After a dose-escalation phase, 18 patients were given the maximum tolerated dose of disulfiram with concurrent copper gluconate while undergoing adjuvant TMZ therapy [13]. Pharmacodynamic activity was analyzed through proteasome assays on peripheral white blood cells. Although the authors caution that 500 mg is generally a well-tolerated dose, certain neurological symptoms can still occur at this dose especially after prolonged drug administration [13]. In addition, this study found that patients within their study did not exhibit any significant difference in survival when referenced against other clinical trials that used TMZ alone after chemoradiotherapy as a historical control [68,69]. The median PFS was 4.5 months (95% CI 0.8–8.2). The median OS was 14.0 months (95% CI 8.3–19.6), and the 2-year OS was 24%. However, the study was not designed to evaluate efficacy of treatment [13].

To better investigate the efficacy of disulfiram as a potential anti-GBM agent, Huang et al. conducted a Phase II open-label single-arm study. Twenty-one patients with GBM demonstrating recurrence and progression after initial RT and TMZ treatment were recruited for the study [14]. The primary endpoint of the trial was based on the overall response rate to assess whether disulfiram and copper gluconate could re-sensitize recurrent GBM patients who had become resistant to TMZ treatment [14]. Secondary endpoints included safety, PFS, and OS. Patients received disulfiram and copper gluconate in addition to TMZ. While it was demonstrated that concomitant disulfiram, copper gluconate, and TMZ was well tolerated, the primary endpoint of 20% overall response rate was not reached and the study was terminated. The median PFS was 1.7 months, and median OS was 7.1 months. The authors noted, however, that about 14% of patients exhibited clinical benefits with prolonged stable disease for more than 6 months suggesting that the combination of disulfiram and copper gluconate had a modest clinical benefit for a small subset of the patient population [14]. It is important to note that the study excluded patients with mutated isocitrate dehydrogenase (IDH) from the study to decrease the heterogeneity rate of the cohort. Therefore, since the results from this trial suggested limited activity against IDH-wild type GBM tumors, Huang et al. proposed that future trials include patients with unselected IDH-wild type GBM which may lead to increased significance [14]. Accounting for the previous limitations, patients are currently being recruited for a Phase I/II clinical trial exploring efficacy of disulfiram and copper as radiosensitizers for GBM therapy (NCT02715609).

### 2.6. Dimethyl Fumarate

Dimethyl fumarate is used in the management of relapsing-remitting multiple sclerosis and psoriasis [15,70]. The drug reduces inflammation and suppresses immune cell function [15]. Recently, studies have found that dimethyl fumarate may render the tumor microenvironment inhospitable to GBM cells by reducing transformed astrocytes and microglia activation [15]. Additionally, it suppresses endothelial cell growth and prevents capillary formation, thereby inhibiting lymphangiogenesis to tumor cells [71]. These properties can make dimethyl fumarate a candidate for GBM treatment.

A Phase I single-arm dose-escalation study was performed to assess the feasibility of combining dimethyl fumarate with TMZ and RT [15]. Twelve patients diagnosed with GBM were treated with varying doses of dimethyl fumarate. The study was successful in demonstrating an acceptable safety profile and establishing a maximum tolerated dose, although the authors noted that it exceeded the FDA-approved dose for multiple sclerosis treatment [15]. The most common adverse events were lymphopenia (58%), decreased CD4 cell count (17%), and thrombocytopenia (17%). The median PFS was 8.7 months with no difference in PFS between those with stable disease or a partial response; median OS was 13.8 months. Future studies are encouraged to justify exceeding the FDA-approved dose and to investigate the efficacy of dimethyl fumarate in treating GBM patients [15].

### 2.7. Sertraline

Sertraline is a selective serotonin reuptake inhibitor (SSRI) that is used to treat depression [8]. It activates postsynaptic neurons by inhibiting uptake of serotonin. SSRIs such as sertraline are considered to be safe and well-tolerated drugs and are capable of achieving therapeutic concentrations when given orally [8]. In vitro studies have suggested that SSRIs have antineoplastic effects against GBM cells when used alone or in combination with drugs like imatinib [28,72]. A Phase I/II proof-of-concept clinical trial was subsequently conducted to investigate safety and efficacy of metronomic TMZ combined with repurposed drugs such as sertraline (NCT02770378). The results of this trial have not been published yet.

### 2.8. Imipramine

Imipramine is an FDA-approved tricyclic antidepressant used in the treatment of severe chronic depression and works by inhibiting serotonin and norepinephrine reuptake [26]. A preclinical study [51] found that imipramine reduced the expression of GSC markers, such as Sox1, Sox2, and CD44. Moreover, the inhibitory effect of imipramine on the human GBM cell line (U87MG) induced autophagy by blocking the PI3K/AKT/mTOR signaling pathway [73]. In fact, only imipramine, among other tricyclic anti-depressants (TCAs), was found to provoke autophagy in glioma cells [51]. Imipramine hydrochloride is currently being investigated in a Phase II trial in patients with recurrent GBM (NCT04863950).

## 3. Drugs of the Alimentary Tract and Metabolism

While there are many drugs in this group that are being investigated to be used for GBM therapy, most trials are still in early phases and the results of these ongoing studies are still pending publication. Of note, two clinical trials investigated sulfasalazine and are discussed within this section. For drugs without published results, we present the current literature and the rationale behind using them to treat patients with GBM.

### 3.1. Metformin

Metformin is a biguanide class of drug that is used to treat type II diabetes mellitus. It acts on mitochondrial complex I in the electron transport chain to inhibit oxidative phosphorylation and causes an increase in the ratio of adenosine monophosphate (AMP) to adenosine triphosphate (ATP) in the cell [74,75]. The accumulation of AMP activates the enzymes fructose 1,6-bisphosphatase, adenylate cyclase, and AMP-activated protein kinase (AMPK), which ultimately reduce gluconeogenesis and increase insulin sensitivity [74]. In addition to its role in metabolism, AMPK also inhibits mammalian target of rapamycin (mTOR) pathway, which is hyper-activated in most tumor cells and plays a role in tumor progression [44,74]. By inhibiting mTOR, AMPK may decrease protein synthesis, cell growth and proliferation, and induce cell cycle arrest and apoptosis [74]. Its antineoplastic effects were supported by epidemiological cohort studies that showed that metformin usage was correlated to lower cancer rates [76]. Moreover, an in vitro conducted by Gritti et al. demonstrated that metformin can inhibit chloride intracellular channel-1 (CLIC1) which can lead to cell cycle arrest of glioma stem cells (GSC) [77]. In addition, in vivo studies have shown that metformin can appreciably cross the blood–brain barrier [78]. Therefore, a number of clinical trials are currently investigating the safety and clinical efficacy of this drug for GBM treatment.

### 3.2. Sulfasalazine

Sulfasalazine is a disease-modifying anti-rheumatic drug (DMARD) used to treat rheumatoid arthritis, ankylosing spondylitis, psoriatic arthritis, ulcerative colitis, and Crohn’s disease [79]. The drug inhibits activation of the nuclear factor kappa B (NF-κB) and the system x_c_^−^ cystine/glutamate antiporter [80,81]. Studies have demonstrated that NF-κB is constitutively active in GBM cells and may contribute to growth and survival of tumor cells [82]. In vitro and in vivo studies have found that sulfasalazine-induced inhibition of NF-κB can inhibit GBM growth [82]. The system x_c_^−^ cystine/glutamate antiporter is the primary pathway for glutamate release and cystine uptake in glioma cells, which promotes chemoresistance, tumor invasion, and tumor growth [17,83]. Furthermore, studies have demonstrated that increased expression of xCT, the light chain subunit of system x_c_^−^ cystine/glutamate, was correlated to poorer prognosis and infiltrative phenotype in patients with GBM [17]. Through inhibition of NF-κB and system x_c_^−^ cystine/glutamate, sulfasalazine demonstrates potential as a drug for GBM.

In a Phase I/II clinical trial, Robe et al. investigated the efficacy of sulfasalazine for treatment of high-grade or recurrent gliomas [16]. Twenty patients diagnosed with GBM who had received conventional treatment with a life expectancy of at least 2 months were enrolled in the study and randomized to receive one of four doses of sulfasalazine [16]. During a scheduled interim analysis of outcome for the first 10 patients for the study, Robe et al. discovered a high incidence of adverse effects and lack of efficacy as indicated by a statistically significant (*p* = 0.005) average growth rate of 2.1 ± 1.7 mL/day that was unrelated to the dose (r = 0.26, *p* = 0.50) [16]. The median PFS was 32 days. Side effects were common, and all patients developed significant adverse events. Two patients died while on treatment or shortly after its discontinuation. The study was terminated, and the authors speculated that tumor growth was unaffected by sulfasalazine treatment due to the small number and large tumor burden of many of the patients. In addition, 87.5% of the tumors tested were unmethylated, a known factor of tumor resistance to alkylating agents. Pharmacokinetics of the drug may vary between mice and humans (intraperitoneal injection in mice vs. oral administration in humans). Moreover, sulfasalazine inhibits only one pathway of NF-κB activation, which may allow gliomas to utilize accessory pathways to achieve NF-κB activation [16].

Takeuchi et al. argued that this previous study recruited patients with advanced disease which may not have accurately reflected the effectiveness of sulfasalazine. They hypothesized earlier sulfasalazine administration may be beneficial [17]. Subsequently, they conducted a clinical trial for patients with newly diagnosed GBM [17]. Twenty-four patients were randomized to receive sulfasalazine, TMZ and RT, or TMZ and RT alone [17]. Similar to the study conducted by Robe et al., they found that sulfasalazine treatment led to a high rate of severe side effects including neutropenia and leukopenia. Since sulfasalazine is considered a safe drug, this suggests that there may be a synergistic effect between sulfasalazine and TMZ which may exacerbate toxic side effects [17]. Moreover, the authors found that sulfasalazine treatment had no significant effect on survival, although it may improve seizure control if an adequate dose could be administered. The median OS, PFS and seizure-free survival (SFS) were 11 months, 4 months, and 7 months, respectively, in the sulfasalazine group, which did not differ from the 13 months, 4 months and 3 months, respectively, observed in the control group. Further investigations are still needed to elucidate the optimal strategy for targeting system xc^−^ and NF-κB in patients with GBM.

### 3.3. Aprepitant

Aprepitant is an antiemetic drug often used for chemotherapy-induced nausea and vomiting [84]. The drug works by blocking substance-P activity through inhibition of neurokinin-1 (NK-1) [84]. Since NK-1 was also found to stimulate growth of GBM [85], in vitro studies were subsequently conducted and showed that aprepitant inhibited GBM growth in a concentration-dependent cytotoxicity pattern, suggesting a potential role as an anti-GBM agent [86].

A Phase II clinical trial titled “Coordinated Undermining of Survival Paths combining 9 repurposed non-oncological drug with metronomic TMZ—version 3” (CUSP9v3) sought to evaluate the safety of a drug combination treatment that included aprepitant, auranofin, captopril, celecoxib, disulfiram, itraconazole, minocycline, ritonavir, and sertraline in combination with TMZ [18]. Ten patients with GBM were treated with uninterrupted TMZ in addition to the 9 drugs listed. The authors found that the multi-drug regimen was generally well-tolerated if individual dose adjustments were made during the trial [18]. The most common adverse events were nausea, headache, fatigue, diarrhea, and ataxia. While this trial was not designed to measure the efficacy of the drugs, Halatsch et al. observed a dichotomy of response to CUSP9v3. While 5 patients progressed quickly and died within 1.5–7 months, 5 other patients did well and had PFS between 12–29 months [18]. The authors suggest that the dichotomy between patient performance may be due to the drug regimen being more effective in patients with low tumor burden or slower growing tumors and suggest that CUSP9v3 may be used in prophylactic maintenance after first-line therapy [18]. Future experiments may include higher-powered studies to evaluate efficacy of these treatment regimens.

## 4. Anti-Viral Drugs

The potential of antiviral drugs to serve as cancer therapy came under investigation when it was noted that incidences of human immunodeficiency virus (HIV) related cancers decreased significantly among patients receiving HIV treatment [87]. The antiviral drugs under investigation demonstrate potential antineoplastic effects through various mechanisms of action [44]. In this section, we discuss the clinical trials of these drugs as well as the rationale for using them for GBM therapy. While some clinical trials of certain drugs (Lopinavir, Ritonavir, and Valganciclovir) have published results, many others are in early phases.

### 4.1. Ritonavir and Lopinavir

Ritonavir and lopinavir are used in HIV treatment [44]. These protease inhibitors decrease the expression of metalloproteases (MMPs) in astrocytes and microglia which may hinder extracellular matrix remodeling and tumor cell invasion [88]. Preclinical studies have found that these drugs can prevent angiogenesis and proliferation and induce apoptosis in GBM cell cultures [89,90].

An open-label Phase II clinical trial was conducted to investigate the therapeutic potential of the combination of ritonavir and lopinavir. Nineteen patients diagnosed with GBM were given the drugs orally twice daily, however, the study was terminated because of inability to achieve the primary objective of at least 30% of patients meeting 6-month PFS [19]. The 6-month PFS was 11%. A complete response was observed in 1 patient (5%). 3 patients (16%) had stable disease as the best response. Fifteen patients (79%) had progressive disease [19]. The authors noted that the lopinavir, a substrate for P-glycoprotein, has poor CNS penetration, and that while ritonavir may increase that bioavailability, it might not be sufficient for therapeutic dosing [19,91,92]. Additionally, ritonavir/lopinavir may have failed because the treatment strategy was directed to a single protease target, not accounting for the heterogeneity that is a hallmark of GBM tumors. This is especially important given that the study did not exclude patients who had received prior treatment regimens which could increase the proportion of drug-resistance [19]. These limitations could be addressed in future research that utilizes drug delivery for drugs with poor BBB penetration.

### 4.2. Valganciclovir

Valganciclovir is an antiviral most commonly used as a prophylaxis to prevent cytomegalovirus (CMV) infection in patients undergoing solid organ transplant [20]. It has also demonstrated efficacy against herpes simplex viruses, Epstein-Barr virus, varicella-zoster virus, and hepatitis B virus [93]. Of note, human CMV has been proposed to contribute to the establishment and progression of different types of tumors and has been detected in many cohorts of patients with GBM [94,95,96]. This has led to an increasing interest in repurposing valganciclovir as a potential treatment for GBM.

The valganciclovir treatment in patients with GBM in Sweden (VIGAS) study was a Phase II double-blind clinical trial that evaluated the efficacy and safety of valganciclovir as an add-on therapy for treatment [20]. Forty-two patients diagnosed with CMV-positive GBM were randomized to receive valganciclovir or placebo in addition to standard treatment (fractionated RT and TMZ, or physician discretion in the case of recurrent GBM) [20]. The primary endpoint was a reduction in tumor volume in the experimental group at 3 and 6 months post-surgery, while the secondary endpoints were PFS at 6, 12, 18, and 24 months [20]. While certain trends were demonstrated, the VIGAS study failed to demonstrate statistically significant differences in tumor volumes between the experimental and control groups at 3 (3.58 vs. 7.44 cm^3^, respectively, *p*  =  0.2881) and 6 (3.31 vs. 13.75 cm^3^, *p*  =  0.2120) months. Median OS was similar in both groups (17.9 vs. 17.4 months, *p*  =  0.430). However, further explorative analysis demonstrated a prolonged survival correlated with longer periods of valganciclovir intake. Patients who had taken valganciclovir for at least 6 months had an OS of 24.1 months, with all of them surviving beyond a year of diagnosis versus 13.1 months (95% CI, 7.9–17.7, *p*  <  0.0001) in patients receiving valganciclovir for 0 or <6 months, and 13.7 months (95% CI, 6.9–17.3, *p*  =  0.0031) in contemporary controls (*p* = 0.0031)) [20]. The long-term beneficial effects may be due to the virus-specific nature of the drug and the drug’s chronic suppressive effect on the virus, as it cannot eliminate the virus from infected cells. Thus, while short-term treatment may not affect tumor growth, the benefits of long-term treatment may manifest once there is sufficient viral suppression [20]. The authors of the VIGAS study noted that OS may have been a better primary endpoint than tumor volume. Additionally, the VIGAS study did not have sufficient power due to small sample size and lack of stratification for significant prognostic factors [20]. However, since the data of this study demonstrate some long-term survival benefits, more robust, higher-powered clinical trials to further investigate the valganciclovir benefits for GBM therapy are needed [20].

### 4.3. Nelfinavir

Nelfinavir is an antiviral compound used to treat HIV [44]. In addition to inhibiting HIV retroviral protease, nelfinavir has been shown to prevent growth of cancer cell lines through the inhibition of the Phosphatidylinositol-3-kinase (PI3K)/v-akt murine thymoma viral oncogene homolog (AKT)/mechanistic target of rapamycin (MTOR) signal transduction pathway which is activated in several types of cancer [97]. It also exhibits additional antitumor effects through numerous mechanisms, including autophagy, endoplasmic reticulum (ER) stress, cellular vacuolization, apoptotic and non-apoptotic cell death [97]. In vitro studies have demonstrated that nelfinavir-induced ER stress enhances tumor necrosis factor-related apoptosis-inducing ligand (TRAIL)-induced cell death in GBM cells [98]. Moreover, in vitro and in vivo models show that nelfinavir may act to increase sensitivity to RT and TMZ in GBM cells [43]. While many clinical trials involving nelfinavir were terminated early (NCT00694837, NCT00915694), one notable Phase I study conducted by Alonso-Basanta et al. (NCT01020292) successfully established the maximum tolerated dose of nelfinavir in combination with RT and TMZ. No dose-limiting toxicity was noted at 625 mg/twice daily. At 1250 mg twice daily, 3 dose-limiting episodes of hepatotoxicity were noted and one dose-limiting episode of diarrhea. This will help pave the way for future Phase II studies [21].

## 5. Anti-Fungal Drugs

### 5.1. Itraconazole

Itraconazole is a broad-spectrum triazole antifungal that was recently found to have antitumor effects [99]. In an in vitro study, Liu et al. demonstrated that itraconazole arrests GBM tumor growth by redistributing cholesterol in the cells. The drug depletes the plasma membrane, leading to inhibition of the AKT1-mTOR pathway and induction of autophagy [99]. Itraconazole was included in the CUSP9v3 study [18]. Details of the study are elaborated upon in the Alimentary Tract and Metabolism section of our discussion. In summary, further work needs to be done to evaluate the safety and efficacy of itraconazole as a potential treatment option for GBM.

### 5.2. Minocycline

Minocycline is a tetracycline-derived antibiotic that has a wide range of therapeutic properties [22]. Due to its highly lipophilic nature, it easily diffuses across the BBB where it has demonstrated anti-inflammatory and neuroprotective effects against a wide range of neurological disorders [100]. In addition to its antimicrobial properties, minocycline also exerts antitumor effects. It inhibits matrix metalloproteinase expression by microglia, which reduces GBM invasion and expansion [101]. It also induces tumor cell death through autophagy and apoptosis [102]. In fact, in vivo studies have demonstrated that minocycline improves survival in rats implanted with intracranial gliomas [103]. There have been a number of clinical trials examining minocycline’s efficacy as an anti-GBM drug. Many of these studies are still in early phases of investigation. Similar to itraconazole, minocycline was included in the CUSP9v3 study, the results of which are elaborated upon in the Alimentary Tract and Metabolism section of our discussion [18]. Additionally, a Phase I trial conducted by Cohen et al. successfully established a maximum tolerated dose of minocycline (400 mg twice a day with no unexpected toxicities) in combination with RT and bevacizumab in patients with recurrent bevacizumab-refractory high grade gliomas [22].

## 6. Anti-Parasitic Drugs

### 6.1. Chloroquine and Hydroxychloroquine

Chloroquine and hydroxychloroquine are 4-aminoquinoline compounds used for the treatment of several infectious and autoimmune diseases [24]. In particular, chloroquine gets protonated in the low pH environment of lysosomes, concentrating within the lysosome of red blood cells and lowering the acidity and efficacy of the lysosome [104]. Chloroquine demonstrates potential anti-cancer by inhibiting autophagy. The drug triggers degradation of autolysosomes, preventing the production of autophagy-derived energy [104]. Notably, chloroquine readily crosses the BBB which makes it a potential candidate for GBM therapy [24].

There have been clinical trials that examined the efficacy of the addition of chloroquine and hydroxychloroquine to GBM treatment. A Phase III randomized, double-blinded study recruited 30 patients with GBM, all of whom received surgery, chemotherapy in the form of carmustine, and radiotherapy. Subjects in the experimental group received chloroquine while the control group received a placebo drug [23]. The study reported an increase in OS within the experimental group with a median survival of 24 months compared to an 11-month median survival for patients in the control group [23]. At the end of the observation period, 6 patients treated with chloroquine had survived 59, 45, 30, 27, 27, and 20 months, respectively while 3 patients from the control group had survived 32, 25, and 22 months, respectively. Although the difference was not statistically significant, the rate of death was approximately half in patients receiving chloroquine as in patients receiving placebo (hazard ratio, 0.52 [95% CI, 0.21 to 1.26]; *p* = 0.139). Investigators for this trial theorized that chloroquine may enhance the cytotoxicity of other cancer drugs or prevent cancer cells from mutating which renders them incapable of eluding the effects of RT or chemotherapy. The results of this clinical trial increased research and clinical trials that investigated chloroquine as a potential treatment for GBM in tandem with other treatment modalities. [24]. Thirteen patients were enrolled to evaluate dose-escalation, safety, and adverse events of chloroquine when combined with TMZ and RT. The study observed significant adverse events at therapeutic doses commonly used for rheumatoid arthritis treatment, such as blurred vision, QT-prolongation, and blurred vision, with no difference in OS when compared to historical data [24]. Despite adverse events, these were considered common side effects associated with chloroquine use [24].

Similar to the chloroquine trials, 92 patients with GBM were included in a clinical trial that suggested that hydroxychloroquine can potentially be an efficacious addition to the standard GBM treatment regimen [25]. However, high dose toxicity limited the efficacy of the drug and there was no improvement in OS. With the toxicity of both chloroquine and hydroxychloroquine as a confounding issue, the development of lower-toxicity autophagy compounds may be necessary [25].

### 6.2. Mefloquine

Mefloquine is another antimalarial drug with similar anti-tumoral effects to chloroquine in GBM cell lines, with higher potency [105]. A Phase I clinical trial investigated the use of TMZ in combination with adjuvant memantine, mefloquine, and metformin for patients newly diagnosed with GBM [9]. Overall, the study was successful in demonstrating the tolerability of these drugs in combination with TMZ. Drug doses were also established for use in future Phase II studies [9].

### 6.3. Mebendazole

Mebendazole is an anti-parasitic drug used to treat several helminth infections [106]. The drug binds to microtubule subunits in parasites and prevents polymerization of tubulin. This induces structural changes and inhibits parasite growth [106]. Mebendazole exerts antineoplastic effects by preferentially binding to cancer cells and inhibiting microtubule polymerization in a manner similar to certain chemotherapy drugs such as paclitaxel, colchicine, and vincristine [106]. In vivo studies demonstrated that mebendazole effectively increased survival in mouse glioma models [106]. A single center dose escalation safety study was conducted by Gallia et al. on 24 patients with newly diagnosed GBM and mebendazole was found to have long-term safety with acceptable toxicity [26]. 4 patients (at 200 mg/kg) developed elevated alanine aminotransferase and/or aspartate transaminase after 1 month, which reversed with lower dosing or discontinuation. The study also showed a 21-month median OS with 41.7% of patients alive at 2 years and 25% at 3 and 4 years [26].

## 7. Drugs of the Cardiovascular System

### 7.1. Captopril

Captopril, which belongs to the class of angiotensin conversion enzyme inhibitors (ACEI), was the first ACEI to enter the market in 1981 [107]. ACEI drugs prevent the conversion of angiotensin I to angiotensin II, causing blood vessel dilatation and ultimately decreasing blood pressure. Angiotensin II has also been found to affect growth and vascularization of gliomas [107]. A 2008 prospective clinical study with 133 patients found that angiotensin II receptors were expressed in 67% of grade III and IV astrocytoma patients and expressed in 53% of high-grade astrocytoma patients. In addition, this study examined the association between angiotensin I and II expression and survival in astrocytoma patients. The authors found that patients with angiotensin II receptor expression had lower survival rates when compared to those with angiotensin negative tumors, and they concluded that angiotensin II receptors contributed to the high mortality rates in high-grade astrocytoma and these findings suggest that angiotensin II receptors might be potential therapeutic targets for high-grade astrocytomas [107]. In addition, captopril was found to inhibit matrix metalloproteinase-2 (MMP-2), an enzyme that facilitates the rapid spread of gliomas through destroying type IV collagen, one of the structural elements of the extracellular matrix [108].

With regards to the CUSP9v3 study, various researchers have used different sets of the nine drugs: (1) Kast et al., 2013: aprepitant, artesunate, auranofin, captopril, copper gluconate, disulfiram, ketoconazole, nelfinavir, sertraline [109]; (2) Kast et al., 2014: aprepitant, artesunate, auranofin, captopril, celecoxib, disulfiram, itraconazole, sertraline, ritonavir [110]; and (3) Skaga et al., 2019: aprepitant, auranofin, captopril, celecoxib, disulfram, itraconazole, minocycline, quetiapine, and sertraline [111]. The effect of CUSP9v3 and TMZ on GSCs was superior to TMZ monotherapy [9]. There is currently one active clinical trial in Phase II evaluating CUSP9v3 in GBM patients (NCT02770378).

### 7.2. Losartan

Losartan is Angiotensin II receptor blocker used in the treatment of hypertension and diabetic nephropathy [29]. It binds reversibly and competitively to the Angiotensin II type 1 receptor (AT1) in vascular smooth muscle and the adrenal gland. In several glioma models, it has been shown to reduce cell proliferation as well as the number of capillary vessels through reducing levels of proangiogenic factors including VEGF, platelet-derived growth factor (PDGF), and fibroblast growth factor (FGF) [22]. It was reported in a retrospective series that the angiotensin receptor blocker is associated with reduced peritumoral edema. The ASTER study, a randomized, placebo-controlled trial, found that Losartan, although well-tolerated, did not reduce the steroid requirement in newly diagnosed GBM patients treated with concomitant RT and TMZ [27].

## 8. Drugs under the Pre-Clinical Investigation

### 8.1. Tricyclic Antidepressants (Amitriptyline, Clomipramine, and Doxepin)

Tricyclic antidepressants (TCAs) are used in the treatment of depression, anxiety disorders, and neuropathic pain. This family of drugs inhibit the reuptake of norepinephrine and serotonin and increase the amount of neurotransmitter in the synaptic cleft [40]. Walker et al. found an inverse relationship between treatment with TCAs and the occurrence of GBM [112]. While TCAs’ antidepressant effects are well-documented, their antitumor properties are less understood. The primary anticancer mechanism is believed to be through targeting glioma cells’ mitochondria while sparing normal cells [22,28]. In addition to TCA’s synergistic effects with chemotherapeutic agents, their immunomodulation activity may also potentiate the efficacy of immunotherapy [113]. While several preclinical studies have shown the potential of TCAs as adjuvant therapy for GBM, more studies are needed to confirm safety and efficacy in these patients [113].

### 8.2. Selective Serotonin Reuptake Inhibitors (Sertraline, Citalopram, Fluoxetine, Fluvoxamine, Escitalopram, and Paroxetine)

Selective serotonin reuptake inhibitors (SSRIs) are the most commonly prescribed antidepressants. They work by increasing serotonin concentrations at the synaptic clefts, thus activating the postsynaptic neurons. SSRIs have recently gained more attention for potential anti-neoplastic property for use in GBM therapy due to their BBB penetration and favorable safety profile. Preclinical studies have shown that SSRIs can inhibit cell proliferation and induce apoptosis in GBM [28]. Fluvoxamine can reduce actin polymerization by inhibiting actin polymerization-related proteins, thus reducing GBM cell invasion [30]. In contrast to fluvoxamine, fluoxetine can induce cell death in gliomas. Moreover, fluoxetine is not toxic to primary neurons and astrocytes [114] and was found to be comparable to TMZ in inhibiting GBM growth in vivo [31]. A preclinical study reported the synergistic effect of fluoxetine, perphenazine, or sertraline with the tyrosine kinase inhibitor imatinib in inhibiting GBM cell proliferation, which suggested potential therapeutic utility for GBM [72].

### 8.3. Benzodiazepines

Benzodiazepines are commonly used in patients with anxiety disorders, sleep disorders, spasticity, status epilepticus, and detoxification and often utilized in general anesthesia. They facilitate the γ aminobutyric acid A (GABA_A_) complex action in the CNS through increasing the frequency of Cl^−^ channel openings [32]. In patients with GBM, diazepam was found to help in post-cancer therapy anxiety and chemotherapy-induced delayed emesis and recent evidence showed an anti-proliferative effect of diazepam in human GBM cells [32]. Studies found that diazepam can induce a cell cycle arrest at the G0/G1 phase in human GBM cells in a dose-dependent pattern [33]. However, although benzodiazepines easily cross the BBB and have anti-proliferative effect, the need for a higher therapeutic dose remains a safety concern.

### 8.4. Repaglinide

Repaglinide is an FDA-approved oral insulin secretagogue used for patients that have type 2 diabetes mellitus [34]. It reduces postprandial glucose excursions by promoting early-phase insulin release from beta-islet cells in the pancreas [34]. It has also been shown to inhibit proliferation and migration of tumor cells through downregulating the expression of anti-apoptotic proteins such as B-cell lymphoma (Bcl-2), Beclin-1 and programmed death-ligand 1 (PD-L1) which eventually leads to apoptosis and autophagy of these cells [34]. Repaglinide was found to prolong survival in mice with GBM and to have significant in vitro cytotoxicity on the human GBM cell line LN229 [34]. Of note, Repaglinide has a fast absorption and short half-life (60 min) [115]. Therefore, future application in the treatment for GBM may consider local intracranial delivery or incorporating the free drug with other vehicles that can extend its duration of action.

### 8.5. Ciglitazone

Ciglitazone is an anti-hyperglycemic agent in the thiazolidinedione class used in the treatment of diabetes mellitus [116]. In addition, it has anti-inflammatory and anti-angiogenic activity through increase of the nuclear factor-kappa B-mediated pathways and reducing vascular endothelial growth factor (VEGF) production, respectively [116]. It was also found to inhibit growth of melanoma cells through the inhibition of chemokine ligand 1 (CXCL1) [116]. In the management of GBM, Ciglitazone is believed to cause loss of the mitochondrial membrane potential in cancer cells which leads to cytochrome c induced apoptosis [117]. Furthermore, it can lead to an increase of reactive oxygen species (ROS) production and lead to apoptosis. Several studies have demonstrated these effects on both rat and human cell lines in vitro, however, some unexpected dose-dependent effects were observed which requires further investigation in order to establish a thorough safety profile [116].

### 8.6. Ibudilast

Ibudilast is an orally bioavailable inhibitor of phosphodiesterase (PDE). It has anti-neuroinflammatory, vasorelaxant, bronchodilator, analgesic, neuroprotective, and possible anti-tumor activities [118]. It is currently approved for use in Japan for the treatment of asthma and post-stroke dizziness [118]. While its mechanism of action for GBM therapy is not completely understood, it is believed to decrease the expression of macrophage migration inhibitory factor (MIF) and its receptor cluster of differentiation 74 (CD74) [35]. Additionally, it inhibits the proto-oncogene tyrosine-protein kinase Src and protein kinase B (Akt) [119]. Since it is known to readily penetrate the BBB, combine synergistically with TMZ, and have no known adverse side effects, [35] Ibudilast has an early phase clinical trial underway for its use in GBM therapy [35].

### 8.7. Amlexanox

Amlexanox is an anti-aphthous ulcer drug. It works by inhibiting the synthesis and release of inflammatory mediators like leukotrienes and histamines from mast cells, neutrophils, and mononuclear cells. It is also a leukotriene dopamine receptor 4 (D4) antagonist and PDE inhibitor. In GBM, it inhibits cell growth through the activation of the Hippo pathway via the downregulation of inhibitor of nuclear factor kappa B kinase subunit epsilon (IKBKE) which leads to apoptosis [36]. In addition, it showed synergistic effect with TMZ in a patient-derived GBM xenograft (PDX) and demonstrated good BBB penetration [120,121]. Currently, Amlexanox is limited to a few studies on GBM cell lines, and its safety profile has not yet been well-established.

### 8.8. Ivermectin

Ivermectin is an FDA-approved drug used in the treatment of parasitic worm infection and rosacea [37,122]. It inhibits several intracytoplasmic and intramitochondrial pathways [123]. Its mechanism of action in GBM cells is not well understood but it is proposed that it induces mitochondrial dysfunction and oxidative stress, inhibits capillary network formation and proliferation, and reduces microRNA 21 (miR-21) levels leading to cell apoptosis [124]. Studies using U87 and T98G GBM cell lines showed efficacy of Ivermectin against these lines [123]. Although Ivermectin has limited BBB penetrability, several studies suggest that it can penetrate the BBB at therapeutic concentrations [123].

### 8.9. NSAIDs

Nonsteroidal anti-inflammatory drugs (NSAIDs) are commonly used as anti-inflammatory, antipyretics, and analgesic agents. They inhibit arachidonic acid pathway through primarily targeting cyclooxegenase-2 (Cox-2) enzyme, and hence decreasing the production of various prostaglandins implicated in a wide range of diseases such including cancer [125]. Previous studies found that prostaglandin E2 (PGE2) can facilitate tumor cell proliferation and angiogenesis while downregulating immune responses and inhibiting apoptosis [126]. Diclofenac and Ibuprofen were found to decrease proliferation and migration of GBM cells [38,39]. Both of these drugs have demonstrated efficacy in reducing phosphorylation of signal transducer and activator of transcription 3 (STAT3). However, diclofenac has an advantage of reducing c-myc expression, extracellular lactate, and lactate dehydrogenase. Therefore, diclofenac can lead to decreased lactate-mediated immunosuppression in gliomas thus stimulating the local immune system. In addition, Ibuprofen needs to be delivered in a high concentration to produce a prolonged therapeutic effect [24].

### 8.10. Ciprofloxacin

Ciprofloxacin is a quinolone antibiotic theorized to work by targeting DNA gyrase, a bacterial enzyme, while also stabilizing DNA strand damage created by DNA gyrase and topoisomerase IV [40]. In GBM, it leads to an increase in the Bcl-2 associated protein X (Bax)/Bcl-2 ratio, which leads to apoptosis induction. Early findings suggest that it could work as an adjuvant for tumor therapy without adverse effect on normal cells [40]. However, efficacy of Ciprofloxacin is not consistently demonstrated across GBM lines [40].

### 8.11. Fluphenazine and Perphenazine

Fluphenazine and Perphenazine are phenothiazine-class antipsychotics used in the treatment of schizophrenia and bipolar disorders [127]. They work as antagonists of postsynaptic D2 receptors in mesolimbic, nigrostriatal, and tuberoinfundibular neural pathways [128]. In GBM they are believed to induce apoptosis through an unknown mechanism [41]. They have been shown to be effective both alone and when combined with TMZ and RT [8,129]. Moreover, Perphenazine was found to have synergistic anti-proliferative effects on GBM cells combined with Imatinib [72]. These drugs are inexpensive, already widely used, and easily penetrate the BBB [129].

### 8.12. Ribavirin

With its discovery in 1972, ribavirin, originally intended to treat Hepatitis C as an antiviral medicine, had shown promise as a possible form of treatment for cancers [130,131]. Its antineoplastic mechanisms include the inhibition of eukaryotic initiation factor 4E (elF4E) and zeste homolog 2 (EZH2). High levels of eIF4E are associated with higher tumor proliferation rate while EZH2 contributes to GBM resistance to radiotherapy. In addition, ribavirin has been found to block the extracellular regulated protein kinases (ERK) and activated protein kinase (MAPK) which are important in neoplasms [131].

In vitro, ribavirin was found to inhibit cell proliferation, increase apoptosis, and prevent the migration of glioma cells. In vivo, the GBM-implanted rodents treated with ribavirin had a significantly longer median survival (19 days) compared to those in the untreated control group (12 days). When ribavirin was combined with TMZ, the median survival rate increased to 29 days [132]. Ochiai et al. tested a triple combination of ribavirin, TMZ and interferon beta (IFN-β) in glioma cell cultures and found that the triple therapy was associated with increased apoptosis and inhibition of cellular growth when compared to the untreated control group, TMZ alone, and TMZ combined with IFN-β [131]. Moreover, ribavirin was found to have an effect on other brain tumors. A recent study found that ribavirin was able to reduce proliferation rates of medulloblastoma cells and to extend survival of medulloblastoma models implanted in animals [133].

## 9. Current Limitations and Recent Advances 

### 9.1. Glioblastoma Stem Cells (GSCs)

Glioblastoma stem cells (GSCs) are a small subpopulation within GBM tumors that have been demonstrated to be responsible for tumor recurrence. In contrast to differentiated GBM cells (d-GCs), GSCs have unlimited self-renewal ability [43], multi-lineage differentiation potential [43,134] and can recapitulate the parental tumor cells in their complex biological nature [8]. While chemotherapies typically kill the proliferating cells, they do not have the same effect on stem cells. These cells remain at the original site after resection, driving tumor recurrence and resistance to standard therapies [134]. The complete elimination of this subpopulation of cells remains a challenge for GBM treatment [135].

In order to repurpose drugs that selectively kill GSCs and limit their off-target adverse effects, it is important to identify biochemical properties unique to the GSCs [136] or metabolic pathways that can specifically target GSCs while sparing surrounding non-malignant cells [137].

Targeting the mitochondrial metabolic pathway is an example of drug repurposing for GBM treatment. Previous studies have described a well-known metabolic phenomenon of cancer cells, known as the Warburg effect [138], which restricts aerobic (oxidative/mitochondrial) metabolism and increases anaerobic metabolism. However, it was found that mitochondrial function remains an essential element for the survival of GSCs and maintenance of their stem-like properties [139]. This led Datta et al. [140] to recently investigate the effect of mitochondrial inhibitors on GSCs. First, they found that classical mitochondrial inhibitors; namely antimycin A, oligomycin A and rotenone, reduce GSCs viability about 100-fold more potently than TMZ. However, since these inhibitors are generally used for preclinical laboratory experiments and have not been evaluated in humans, they subsequently screened 1600 clinically tested and FDA-approved drugs for their mitochondrial inhibitory properties and identified three drugs; mitoxantrone, trifluoperazine and pyrvinium pamoate. Their work eventually demonstrated that these three FDA-approved drugs can decrease GSC viability about 50-fold more potently than TMZ.

Vargas-Toscano et al. [141] recently demonstrated that blocking neurotransmitter signaling pathway can have the potential to suppress GSCs proliferation and survival. The authors demonstrated that trihexyphenidyl, an acetylcholine receptor antagonist used for Parkinson’s disease; homatropine, an acetylcholine drug mainly used in ophthalmology; and rizatriptan, used as an anti-migraine agent could effectively kill GSCs [141]. However, it is important to mention that these experiments were in vitro, so translation needs to be further clarified.

Manipulating the Renin-Angiotensin System (RAS) is another example of current research efforts on repurposing drugs targeting GSCs. In fact, multiple early experiments have consistently found that RAS plays an important role in cancers through resisting apoptosis [142,143,144], evading growth suppressors [145], sustaining proliferative signaling, inducing angiogenesis [146,147], as well as regulating inflammation [148,149], cellular migration [150], invasion and metastasis [146,151]. Subsequent research has shown that various components of the RAS are expressed by GSCs as well as the endothelium of the micro-vessels within GBM [152,153], suggesting that modulation of the RAS may provide novel therapeutic potential in GBM [152]. This eventually led many researchers to investigate the efficacy of RAS-modulating drugs in animal and human GBM models. Recently, a reformulated liquid aspirin, IP1867B, an inhibitor of COX1/COX2 and IGF/IGFR-1-signaling pathways, was found to reduce high-grade glioma tumor burden. This is believed to be due its effect on GSCs [154]. More recently, Skaga et al. [111] reported the efficacy of a coordinated pharmacological blockade in GSCs derived from 15 GBM patients using the coordinated CUSP-9v3 strategy. They found that in clinical plasma concentrations, the effect of the CUSP9v3 with TMZ was superior to TMZ monotherapy. Moreover, the CUSP9v3 significantly reduced Wnt-activity; an important signaling pathway in GSC. 

Important mechanisms of GBM resistance include tumor plasticity, genetic evolution, and clonal selection [155]. GBM plasticity results from the dynamic equilibrium between undifferentiated GSCs and differentiated non-GSCs. The differentiated non-GSCs has the potential to revert (dedifferentiate) into GSCs due to epigenetic alteration or factors in the tumor microenvironment [155]. In vitro and in vivo studies have found that exposure of differentiated GBM cells to therapeutic doses of TMZ or ionizing radiation increases the GSC pool [156]. Moreover, a recent study analyzed longitudinal genomic and transcriptomic data from 114 patients with GBM and found that 63% of these patients experienced expression-based subtype changes after exposure to therapy [157]. With the modest improvements in the outcomes of GBM patients over the past few decades, it will be critical to look for new treatment strategies that target CSCs when considering drug repurposing for GBM. It is especially important to identify the transcriptional programs that endow these GCSs with self-renewal and chemoresistant properties [158,159].

### 9.2. Overcoming the Blood-Brain-Barrier in Drug Repurposing for GBM

The BBB is considered a major challenge to repurposing drugs to be used for GBM therapy. Several recent advances in drug delivery technology have provided promising strategies to overcome this challenge [160,161]. These drug delivery strategies can be broadly divided into local and systemic groups.

Several investigators have argued that high grade gliomas, including GBM, can induce major alterations of the normal vascular function resulting in a disrupted, leaky blood–brain tumor barrier (BBTB) as manifested by contrast-enhanced MRI [162,163]. This leaky BBTB was initially postulated to increase the efficacy of systemically administered drugs [162]. However, recent studies have demonstrated that it is unlikely that this local disruption is sufficient to allow intratumoral drug penetration in therapeutic quantities [164]. Moreover, the disruption of the BBB in GBM is significantly heterogeneous and tumor cells can be found in areas with an intact BBB. This is especially evident in the invasion areas of the tumor which typically do not enhance on contrast-enhanced MRI [165,166,167,168].

**Figure 1:**
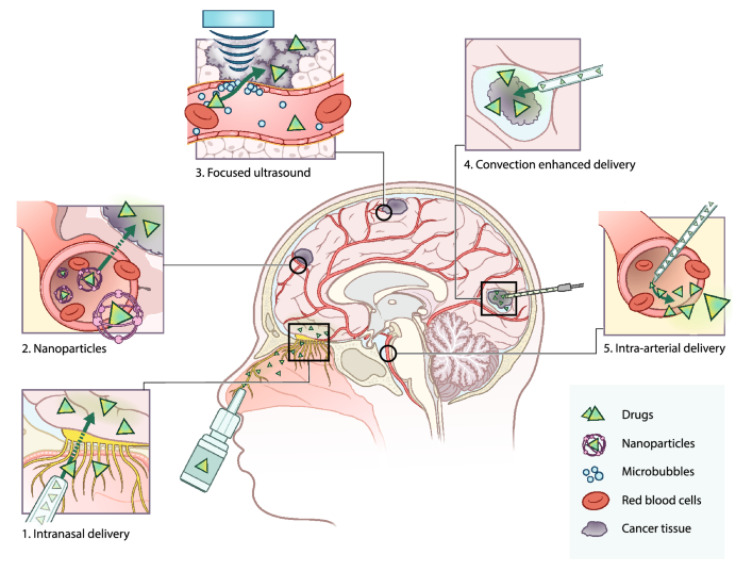
Overview of current drug delivery methods for the treatment of primary brain tumors. Panel 2—nanoparticles: nanoparticles encapsulate drugs to increase plasma half-life and allow entry to the brain parenchyma by the enhanced permeability and retention (EPR) effect, endocytosis, and receptor-mediated transcytosis. Panel 3—microbubble-mediated focused ultrasound: microbubbles are intravenously administered and upon the application of focused ultrasound, microbubbles start to oscillate. The oscillation disrupts the BBB, temporarily opening it to allow drugs to enter the brain parenchyma. Panel 4—convection enhanced delivery (CED): surgical placement of catheters in the brain to administer the drug directly in the tumor site [172].

To enhance therapeutic uptake in tumors, researchers have been developing strategies to modulate BBB permeability Figure 1. Nanoparticle drug delivery is one strategy that can enable these drugs to cross the BBB and reach therapeutic concentrations in the brain [169]. For example, Doxorubicin-loaded nanoparticles have demonstrated significant in vivo efficacy in GBM models [169]. Selective disruption of the BBB using high intensity focused ultrasound (HIFU) is another strategy that can allow for therapeutic concentrations in the tumor bed [170].

Intratumoral drug delivery for GBM treatment involves the local administration of therapeutic molecules through direct injection at the tumor site [171] (Figure 1) [172]. One of the main advantages of this technique is the ability to achieve high drug concentration at the site of tumor or tumor resection while simultaneously reducing systemic drug exposure. This strategy minimizes systemic toxicity and side effects and can be considered as a complementary addition to resection surgery [173].

Local delivery of anti-tumor agents are valuable in targeting GBM cells due to the invasive nature of this tumor [174]. Multiple studies are investigating various delivery materials and optimizing formulations for drug delivery. Increased efforts are focused on the biodistribution and kinetics of drug release as well as the biocompatibility and biodegradability of the vehicles best suited for intracranial delivery [160,161,175]. Gliadel^®^ wafers represent an early and successful example of local intracranial drug delivery that resulted in extending the survival of patients with newly diagnosed and recurrent GBM [173,176].

It is important to note that drug repurposing involves other non-academic disciplines to address the technical, medical, regulatory and marketing hurdles for development and commercialization [177]. For instance, a limiting factor in repurposing drugs for GBM can be the lack of interest from the pharmaceutical companies due to the limited revenue from generic drugs when compared to novel drugs. This is especially likely since GBM is considered a relatively rare disease when compared to other common diseases [177]. In addition, certain legal and regulatory issues can hinder the new commercialization of repurposed drugs [177].

## 10. Conclusions

A wide range of drugs that are already in clinical use have been found to target GBM-associated signaling pathways. Many of these drugs are being repurposed for use in the treatment of GBM patients and are currently in the clinical trial phase. Others have demonstrated possible mechanisms of action in GBM and are in pre-clinical investigation. Since GSCs have been found to be a main source of tumor recurrence after surgery, recent studies have investigated molecular pathways targeting this cell population. Repurposing drugs to target these pathways can be a potential strategy to counteract tumor recurrence. While several repurposed drugs have shown significant efficacy against GBM cell lines, the BBB can hinder them from reaching intratumoral therapeutic concentrations. Systemically delivering these drugs by nanoparticles is one strategy that can enable them to cross the BBB. Alternatively, localized intracranial delivery can help to achieve high drug concentration at the site of tumor resection while simultaneously minimizing deleterious systemic effects. The repurposing of approved drugs for their new use in GBM therapy has promising potential.

## Figures and Tables

**Table 1:** Drugs in the clinical trial phase. Acronyms used: AKT: v-akt murine thymoma viral oncogene homolog; ALDH: aldehyde dehydrogenase; CMV: cytomegalovirus; FGF: fibroblast growth factor; GBM: glioblastoma; HDAC: histone deacetylases; MGMT: O6-methylguanine-DNA methyltransferase; MMP-2: matrix metalloproteinase-2; mechanistic target of rapamycin; NMDA: N-methyl-D-aspartic acid; PDGF: platelet-derived growth factor; PI3K: Phosphatidylinositol-3-kinase; PON: paraoxonase; ROS: reactive oxygen species; RT: radiation therapy; TGF-β: transforming growth factor-β, and VEGF: vascular endothelial growth factor.

Drug	Primary Indication	Mechanism of Action in GBM	Clinical Trials
Memantine	Alzheimer’s Disease	NMDA antagonist Enhances cell death Alters morphological features of tumor cells Inhibits migration and division of tumor cells	Phase I clinical trial [9]
Levetiracetam	Epilepsy	Inhibits cell growth and proliferation and increases autophagy Increases GBM cells sensitivity to TMZ and RT Inhibits HDAC Downregulates MGMT Increases p53 expression	Retrospective survival analysis [10]
Valproic Acid	Epilepsy and bipolar disorders	Reduces PON2 expression in cells Increases ROS production Induces Bim production PON2-Bim cascade inhibits GBM progression Induces G2/M cell cycle arrest	Open-label Phase II clinical trial [11] and Phase II study [12]
Disulfiram	Alcohol abuse	Inhibits tumor growth by inhibiting ALDH Diethyldithiocarbamate, a metabolite of disulfiram, chelates with Cu and zinc ions to form complexes that inhibit proteasomes and increase cytotoxicity through accumulation of oxygen free radicals	Phase I open-label [13] and Phase II open-label single-arm study [14]
Dimethyl Fumarate	Multiple sclerosis and psoriasis	Renders the tumor microenvironment inhospitable to GBM cells by reducing transformed astrocytes and microglia activation Suppresses endothelial cell growth and prevents capillary formation to tumor cells	Phase I single-arm dose-escalation study [15]
Sertraline	Depression and other psychiatric disorders	Reduces tumor growth	Phase I/II proof-of-concept trial to investigate safety and efficacy of metronomic TMZ combined with repurposed drugs (NCT02770378).
Imipramine	Severe chronic depression	Reduces the expression of GSCs markers such as Sox1, Sox2 and CD44 Induces autophagy by blocking PI3K/AKT/mTOR signaling pathway	Phase II trial in patients with recurrent GBM (NCT04863950).
Metformin	Type II diabetes mellitus	Activates AMPK and inhibits mTOR Decreases protein synthesis, cell growth and proliferation, and induces cell cycle arrest and apoptosis	Multiple clinical trials underway (NCT02780024, NCT03243851, NCT04691960, NCT03151772, NCT01430351, NCT04945148, NCT02149459)
Sulfasalazine	Autoimmune diseases including rheumatoid arthritis	Inhibits activation of the nuclear factor kappa B (NF-κB) and the system x_c_^−^ cystine/glutamate antiporter and thus inhibits tumor growth	Phase I/II clinical trials [16,17]
Aprepitant	Chemotherapy-induced nausea and vomiting	Concentration-dependent cytotoxicity through blocking substance-P and neurokinin-1	Phase II clinical trial titled “Coordinated Undermining of Survival Paths combining 9 repurposed non-oncological drug with metronomic TMZ—version 3” (CUSP9v3) [18]
Ritonavir and Lopinavir	HIV	Decreases the expression of MMPs in astrocytes and microglia Hinders extracellular matrix remodeling and tumor cell invasion	Open-label Phase II clinical trial [19]
Valganciclovir	Prevention of CMV infection in patients undergoing solid organ transplant	Human CMV has been proposed to contribute to the establishment and progression of different types of tumors and has been detected in many cohorts of patients with GBM	Phase II double-blind clinical trial (VIGAS) [20]
Nelfinavir	HIV	Prevents growth of cancer cell lines through the inhibition of the PI3K/AKT/ MTOR signal transduction pathway	Phase I trial [21]
Itraconazole	Fungal infections	Arrests GBM tumor growth by redistributing cholesterol in the cells Depletes the plasma membrane Inhibits the AKT1-MTOR pathway and induce autophagy	Phase IIb/IIa proof-of-concept study as part of CUSP9v3 [18]
Minocycline	A tetracycline-derived antibiotic that has a wide range of therapeutic properties	Inhibits matrix metalloproteinase expression by microglia, which reduces glioma invasion and expansion. Induces tumor cell death through autophagy and apoptosis.	Phase I trial [22] (CUSP9v3) [18]
Chloroquine and hydroxychloroquine	Malaria	Induces autophagy/reduction of cell proliferation/inhibition of MMP-2 activity and cell invasion/inhibition of TGF-β secretion and signaling pathway	Phase III randomized, double-blinded study [23] & Phase I/II trials [24,25]
Mefloquine	Malaria	Inhibits proliferation and induction of cell cycle arrest in G2/M phase through enhancement in p21WAF1/CIP1 and p53 expression/induction of autophagy	Phase I clinical trial [9]
Mebendazole	Nematodes (round worm) infections	Disrupts microtubule formation Inhibits microtubule polymerization Inhibits protein kinase Induces metaphase arrest	Single center dose escalation safety study [26]
Captopril	Hypertension and diabetic nephropathy	Reduces cell proliferation and vascularization of the tumor Limits tumor invasion through inhibiting MMP-2	Phase II clinical trial evaluating CUSP9 in GBM patients (NCT02770378).
Losartan	Hypertension and diabetic nephropathy	Reduces cell proliferation as well as the number of capillary vessels, through reducing levels of proangiogenic factors including VEGF, PDGF, and FGF [22]	ASTER study [27]

**Table 2:** Drugs in preclinical investigation. Acronyms used: AKT: v-akt murine thymoma viral oncogene homolog; AMPK: adenosine monophosphate-activated protein kinase; Bcl-2: B-cell lymphoma-2; Beclin-1: Beclin-1 protein; CD74: cluster of differentiation 74; DCA: dichloroacetate; elF4E: eukaryotic initiation factor 4E; ERK: extracellular regulated protein kinases; EZH2:zeste homolog 2; FAK: focal adhesion kinase; FDA: food and drug administration; GBM: glioblastoma; GSC: glioma stem cells; IKBKE: inhibitor of nuclear factor kappa B kinase subunit epsilon; MAPK: mitogen-activated protein kinase; MIF: migration inhibitory factor; miR-21: microRNA 21; MTOR: mechanistic target of rapamycin; PI3K: Phosphatidylinositol-3-kinase; PD-L1: programmed death-ligand 1; ROS: reactive oxygen species; STAT3:signal transducer and activator of transcription 3; and TMZ: temozolomide.

Drug	Primary Indication	Possible Mechanisms of Action in GBM
Tricyclic antidepressants (amitriptyline, clomipramine, and doxepin)	Depression, anxiety disorders, and neuropathic	Reduces cell proliferation, and induce autophagy by inhibiting PI3K/Akt/mTOR signaling pathway Reduces cell stemness, and regulate GSC plasticity Limits cell invasive capacity Potentiates the efficacy of immunotherapy [28,29]
Selective serotonin reuptake inhibitors (sertraline, citalopram, fluoxetine, fluvoxamine, escitalopram, and paroxetine)	Depression, bipolar and anxiety disorders	Inhibit GBM proliferation invasion, and increase apoptosis through: Inhibits polymerization of actin Lamellipodia suppression Decreases FAK, Akt and mTOR phosphorylation Increases Ca^2+^ influx into mitochondria Releases proapoptotic factors (cytochrome c and caspases enzymes) [28,30,31]
Benzodiazepines	Anxiety disorders, sleep disorders, spasticity, status epilepticus, and detoxification and often utilized in general anesthesia	Induces cell cycle arrest at the G0/G1 phase in a dose-dependent pattern Helps patients with post-cancer therapy anxiety and chemotherapy-induced delayed emesis [32,33]
Repaglinide	Type 2 diabetes mellitus	Inhibits proliferation and migration of tumor cells through downregulating the expression of anti-apoptotic proteins such as: Bcl-2, Beclin-1 and (PD-L1) [34]
Ciglitazone	Type 2 diabetes mellitus	Causes loss of the mitochondrial membrane potential in cancer cells which leads to cytochrome c induced apoptosis Leads to an increase of ROS production and eventually leading to cell death [6]
Ibudilast	Asthma and post-stroke dizziness	Decreases the expression of macrophage MIF and its receptor CD74 [35]
Amlexanox	Aphthous ulcer	Inhibits cell growth through the activation of the Hippo pathway via the downregulation of IKBKE which leads to apoptosis Shows synergistic effect with TMZ [36]
Ivermectin	Parasitic worm infection and rosacea	Induces mitochondrial dysfunction and oxidative stress Inhibits capillary network formation and proliferation, and reduces miR-21 levels leading to cell apoptosis [37]
NSAIDs	Anti-inflammatory, antipyretics, and analgesic agents	Decreases proliferation and migration of GBM cells through inhibiting arachidonic acid pathway metabolites (prostaglandin E2) and reducing phosphorylation of STAT3 Diclofenac has an advantage of reducing c-myc expression, extracellular lactate, and lactate dehydrogenase and therefore, diclofenac can lead to decreased lactate-mediated immunosuppression in gliomas thus stimulating local immune system [38,39]
Ciprofloxacin	Antibiotic for bacterial infections	Increases the Bcl-2 associated protein X (Bax)/Bcl-2 ratio, which leads to apoptosis induction [40]
Fluphenazine and Perphenazine	Schizophrenia and bipolar disorders	Induces apoptosis through an unknown mechanism [41]
Ribavirin	Antiviral drug used for Hepatitis C infection	Inhibits elF4E and EZH2. High levels of eIF4E are associated with higher tumor proliferation rate while EZH2 contributes to GBM resistance to radiotherapy. Blocks the ERK and MAPK which are important in neoplasms [41]
Chloramphenicol	Antibiotic for bacterial infections	Inhibits aldehyde dehydrogenase which leads to GSC dysfunction [42]
Phenformin	Type 2 diabetes mellitus	Inhibits tumor growth, cell self-renewal and reduce cell stemness and mesenchymal markers though binding to AMPK Has a synergistic effect with TMZ and DCA in targeting GSCs Discontinued by the FDA in the 1970s due to its lactic acidosis [43]

## Data Availability

Not applicable.
